# Association between brain-derived neurotrophic factor and von Willebrand factor levels in patients with stable coronary artery disease

**DOI:** 10.1186/s12872-018-0762-z

**Published:** 2018-02-06

**Authors:** Hong Jin, Yifei Chen, Bilei Wang, Yi Zhu, Long Chen, Xiqiong Han, Genshan Ma, Naifeng Liu

**Affiliations:** 1 Department of Cardiology, Zhongda Hospital, Medical School of Southeast University, Nanjing, Jiangsu 210009 China; 2 Department of Cardiology, Wuxi Xishan People’s Hospital, Wuxi, Jiangsu 214000 China

**Keywords:** Brain-derived neurotrophic factor, Coronary artery disease, Von Willebrand factor, Prognosis

## Abstract

**Background:**

Brain-derived neurotrophic factor (BDNF) is a neurotrophin involved in angiogenesis and maintenance of endothelial integrity. Whether circulating BDNF levels are associated with von Willebrand factor (vWF) levels, which are indicators of endothelial dysfunction is not known. This study investigated the association between plasma BNDF and vWF levels and whether these biomarkers could predict cardiovascular events at a 12-month follow-up in patients with stable coronary artery disease (CAD).

**Methods:**

We recruited 234 patients with suspected angina pectoris. Subjects were divided into CAD (*n* = 143) and control (*n* = 91) groups based on coronary angiography. Plasma BDNF and vWF levels were measured using ELISA. Patients were followed-up for one year, and information on adverse cardiac events was collected.

**Results:**

CAD patients exhibited significantly lower plasma BDNF and higher vWF levels than those of control patients. High vWF levels were associated with low BDNF levels even after adjustment for age, gender, low-density lipoprotein (LDL) levels, and the presence of diabetes mellitus. A receiver operating characteristic curve was used to determine whether low BDNF and high vWF levels could predict adverse cardiovascular events. The area under the curve for vWF and the inverse of BDNF were 0.774 and 0.804, respectively.

**Conclusions:**

These findings suggest that endothelial dysfunction is an important determinant of the impaired circulating BDNF levels, and they further reflected cardiovascular prognosis in stable CAD patients.

## Background

Brain-derived neurotrophic factor (BDNF) is a neurotrophin, and it promotes survival, differentiation, and maintenance of neurons [1]. However, increasing evidence suggests that BDNF plays a pivotal role in the cardiovascular system. Recent studies demonstrated that BDNF and its receptors (Tyrosine receptor kinase B, TrkB) are also expressed in the peripheral vasculature, where it stimulates angiogenesis, promotes the survival of endothelial cells and maintains vascular integrity [2–4]. BDNF is also present in blood [5]. Decreased plasma BDNF levels have been reported in patients with or at risk for cardiovascular disease [6–8]. Moreover, low serum BDNF levels in patients with coronary artery disease (CAD) were associated with an increased risk of adverse cardiovascular events and mortality [9]. In contrast, high BDNF levels in a large community-based cohort were also prospectively associated with a decreased risk of cardiovascular disease and mortality [10].

Numerous parameters influence circulating BDNF levels. Lower BDNF concentrations have been associated with known risk factors for CAD including lipid levels, elderly age, male gender, smoking, diabetes mellitus, and physical inactivity [9, 11–13]. Additionally, preclinical evidence showed that cardiovascular BDNF is primarily localized within endothelial cells and circulating BDNF levels may be an index of endothelial BDNF expression in animal model [14]. Indeed, endothelial BDNF expression was impaired in hypertensive rats [15]. Recent clinical studies have also identified a potential link between circulating BDNF levels and endothelial dysfunction, although the data were limited to metabolic syndrome [16] and hypertension [17]. Therefore, we hypothesized that endothelial dysfunction is an important factor for low circulating levels of BDNF in patients with CAD.

Endothelial dysfunction plays an important role in the development of atherosclerosis, thereby increasing the risk of severe cardiovascular endpoint events [18]. Endothelial dysfunction may be assessed using changes in circulating markers, such as plasma von Willebrand factor (vWF), which indicate endothelial damage because these markers are primarily produced by endothelial cells to facilitate platelet adhesion and aggregation at sites of injury [19]. Previous studies have reported increased levels of vWF in CAD patients, and high vWF levels may also predict cardiovascular events in these patients [20, 21]. Therefore, circulating vWF levels may be a useful indicator of endothelial dysfunction.

This study examined whether plasma BDNF levels were related to vWF levels in patients with stable CAD and whether these biomarkers could predict adverse cardiovascular events at a 12-month follow-up.

## Methods

### Study design and participants

Patients with suspected angina pectoris were consecutively recruited at admission to our cardiac center from May 2013 to July 2015. Patients underwent coronary angiography voluntarily in the hospital. All participants gave full written informed consent, and the study was approved by the Committee of Clinical Investigation of Southeast University School of Medicine.

CAD was diagnosed using coronary angiography. Two experienced cardiologists visually estimated the degree of coronary stenosis. Patients with > 50% stenosis of the primary coronary artery or its major branches were diagnosed with CAD. Individuals with < 20% stenosis were considered controls. A total of 234 patients were included in our analysis, with 143 patients in the CAD group and 91 patients in the non-CAD group.

Diabetes was diagnosed according to data obtained from medical records. Dyslipidemia was diagnosed when the total fasting serum cholesterol was ≥5.2 mmol/L, triglycerides were ≥1.7 mmol/L or low-density lipoprotein (LDL) cholesterol was ≥2.6 mmol/L according to the Chinese Guidelines for the Prevention and Treatment of Dyslipidemia [22]. Hypertension was diagnosed when systolic blood pressure was at least 140 mmHg and/or diastolic blood pressure was at least 90 mmHg or when patients were taking antihypertensive drugs.

Exclusion criteria included patients with acute coronary syndrome (ACS), heart failure (ejection fraction (EF) < 50%), a history of coronary arterial revascularization, previous myocardial infarction (MI), severe valvular heart disease, idiopathic cardiomyopathy, immune disease, cerebrovascular diseases, aphasia, dementia, depression, schizophrenia, homological diseases, severe liver disease, renal failure, cancers, and a mental disorder or those taking antidepressant drugs or tranquilizers.

### Follow-up

CAD patients were followed in the outpatient clinic after discharge for one year. Adverse cardiac events were identified by searching the medical records and confirmed through direct contact with the patients or relatives. An adverse cardiac event was defined as all-cause mortality, ACS or unplanned coronary revascularisation. ACS was defined as the clinical diagnosis of ST segment elevation myocardial infarction (STEMI), non-STEMI or unstable angina pectoris in accordance with the guidelines of the European Society of Cardiology [23]. Unplanned coronary revascularisation was defined as unplanned repeat percutaneous coronary intervention (PCI) (culprit or non-culprit coronary artery) or coronary artery bypass grafting (CABG).

### Laboratory procedures

Blood samples were obtained from patients in the morning hours following standardized procedures within 24 h of hospitalization. Blood samples were collected in tubes containing EDTA (pH 7.5) and immediately centrifuged at 3000 rpm for 10 min at 4 °C. Plasma samples were stored at 80 °C. Plasma BDNF and vWF levels were measured using commercial enzyme-linked immunosorbent assay (ELISA) kits (BDNF Emax® Immuno Assay System, Promega, USA; IMUBIND® vWF Activity ELISA Kit, American Diagnostica, Stamford, USA). The measurements were performed strictly according to the manufacturer’s instructions. Other biochemical parameters were determined using standard laboratory methods in the routine hospital laboratory.

### Statistical analysis

All data are expressed as the means ± standard deviation for approximately normally distributed data and as the medians (interquartile range) for skewed continuous variables. Comparisons of continuous variables for the two groups were performed using Student’s *t*-tests or Mann-Whitney *U* tests. The chi-square test was used for comparisons of categorical variables between groups. Pearson’s correlation coefficient and the nonparametric Spearman’s correlation method were used for correlation analyses. Variables with skewed distribution were transformed logarithmically before Pearson’s correlation to fulfill the conditions required for this type of analysis. The results are presented as the coefficient of correlation (r). Associations between BDNF levels and key variables were analyzed using univariate and multiple regression analyses. Logistic regression analysis was performed to identify significant parameters for the presence of CAD. Results of binary logistic regression analysis are presented as odds ratios (OR) and 95% confidence intervals (CI). Receiver operating characteristic (ROC) analysis was performed to determine whether BDNF and vWF were predictive of adverse cardiac events. BDNF was transformed reciprocally before ROC analysis because of the inverse relationship between BDNF levels and cardiovascular events. Comparisons of areas under the ROC curves (AUCs) were performed as recommended by DeLong et al. [24]. All tests were two-sided. *P* values less than 0.05 were considered significant. Statistical analyses were performed using SPSS software 19.0 (SPSS Inc., Chicago, IL, USA).

## Results

A total of 143 CAD patients and 91 control patients were enrolled in this study. The mean age of the cohort was 65.9 ± 10.5 years, and 34.6% were women. Table 1 shows a comparison of the clinical characteristics of patients in the two groups. CAD patients were older and more likely to be male compared to the control group. Cardiovascular risk factors, including diabetes mellitus (*p* = 0.006) and hypertension (*p* = 0.001), were more prevalent in the CAD group. There were no differences between the two groups in body mass index, smoking, systolic/diastolic blood pressure, fasting glucose, triglycerides, total cholesterol, LDL cholesterol, high-density lipoprotein (HDL) cholesterol, platelet count, or creatinine. Most patients in the cohort were taking at least one recommended medication [e.g., aspirin, beta-blockers, statin, angiotensin-converting enzyme inhibitors (ACEI)/angiotensin receptor blockers (ARB), or calcium channel blockers (CCB)] at hospital admission. The proportions of patients who used aspirin, beta-blockers, statin, ACEI/ARB, and CCB therapy were 29.1%, 29.1%, 23.5%, 40.6%, and 44.4%, respectively. The rate of use of these recommended medications at hospital admission was similar between groups. Plasma BDNF concentrations in CAD patients [937 pg/ml (679 to 1263)] were significantly lower than those in control patients [1361 pg/ml (884 to 1846)] (*p* < 0.001; Table 1). CAD patients exhibited significantly higher vWF levels (111 ± 27 IU/dl) compared to those of the control group (89 ± 18 IU/dl) (*p* < 0.001; Table 1).

**Table 1:** Clinical characteristics of the study groups (*n* = 234)

	Overall	Stable CAD (*n* **=** 143)	Non-CAD (*n* **=** 91)	*p* value
Age, years	65.9 ± 10.5	67.9 ± 10.3	62.8 ± 10.2	< 0.001
Gender, male, n (%)	153 (65.4)	104 (72.7)	49 (53.8)	0.003
BMI (kg/m^2^)	24.1 ± 2.4	24.2 ± 2.5	23.9 ± 2.4	0.450
Smoking, n (%)	102 (43.6)	68 (47.6)	34 (37.4)	0.125
SBP (mmHg)	136 ± 15	135 ± 15	137 ± 14	0.340
DBP (mmHg)	82 ± 11	81 ± 10	83 ± 12	0.161
Triglycerides (mmol/L)	1.62 (1.23–2.20)	1.65 (1.26–2.20)	1.57 (1.09–2.20)	0.266
TC (mmol/L)	4.54 ± 1.03	4.66 ± 1.07	4.36 ± 0.94	0.031
LDL cholesterol (mmol/L)	2.60 ± 0.83	2.67 ± 0.91	2.49 ± 0.68	0.102
HDL cholesterol (mmol/L)	1.18 ± 0.28	1.15 ± 0.29	1.21 ± 0.26	0.082
Platelet count(× 10^4^/μl)	185 (166–217)	183 (154–216)	196 (167–230)	0.099
Creatinine (μmol/L)	76 (65–87)	76 (66–89)	73 (62–81)	0.067
Glucose (mmol/L)	5.5 (4.9–6.9)	5.7 (5.0–7.2)	5.4 (4.8–6.7)	0.162
Diabetes mellitus, n (%)	79 (33.8)	58 (59.4)	21 (23.1)	0.006
Hypertension, n (%)	156 (66.7)	107 (74.8)	49 (53.8)	0.001
Medication use, n (%)				
Aspirin	68 (29.1)	44 (30.8)	24 (26.4)	0.566
Beta-blocker	68 (29.1)	41 (28.7)	27 (29.7)	0.987
Statin	55 (23.5)	34 (23.8)	21 (23.1)	0.902
ACEI/ARB	95 (40.6)	59 (41.3)	36 (39.6)	0.903
CCB	104 (44.4)	64 (44.8)	40 (44.0)	0.905
CRP (mg/L)	1.17 (0.67–2.20)	1.23 (0.69–2.20)	0.93 (0.61–2.21)	0.079
vWF (IU/dl)	102 ± 26	111 ± 27	89 ± 18	< 0.001
BDNF (pg/ml)	1187 (784–1543)	937 (679–1263)	1361 (884–1846)	< 0.001

Values are shown as the means ± SD, median (interquartile range) or percentage. *CAD* coronary artery disease, *BMI* body mass index, *SBP* systolic blood pressure, *DBP* diastolic blood pressure, *TC* total cholesterol, *LDL* low-density lipoprotein, *HDL* high-density lipoprotein, *ACEI* angiotensin-converting enzyme inhibitor, *ARB* angiotensin receptor blocker, *CCB* calcium channel blocker, *CRP* C-reactive protein, *vWF* von Willebrand factor, *BDNF* brain-derived neurotrophic factor

Univariate logistic analyses revealed that age, male sex, hypertension, diabetes mellitus, triglycerides, HDL cholesterol, BDNF and vWF were significantly associated with the presence of stable CAD (Table 2). These parameters were entered into a multivariate logistic analysis, and only BDNF (HR = 2.590; 95% CI, 1.287–5.215; *p* = 0.008) and vWF (HR = 2.686; 95% CI, 1.424–5.066; *p* = 0.002) were independent predictors of the occurrence of stable CAD (Table 2).

**Table 2:** Logistic regression analyses of cardiovascular risk factors for predicting patients with stable CAD (n = 234)

	Univariate analysis			Multivariate analysis		
	HR	95% CI	*p* value	HR	95% CI	*p* value
Age (per year)	1.048	1.021–1.076	< 0.001	0.995	0.967–1.024	0.732
Male sex	2.286	1.315–3.972	0.003	1.646	0.884–3.064	0.116
Currently smoking	1.520	0.889–2.600	0.126			
Hypertension	2.548	1.457–4.456	0.001	1.121	0.586–2.142	0.730
Diabetes mellitus	2.275	1.260–4.107	0.006	1.578	0.785–3.174	0.201
Body mass index > 23 (kg/m^2^)	0.966	0.557–1.673	0.900			
Triglycerides > 1.7 (mmol/L)	1.260	0.737–2.154	0.398			
Total cholesterol > 5.2 (mmol/L)	1.123	0.585–2.153	0.728			
LDL cholesterol > 2.6 (mmol/L)	1.481	0.845–2.599	0.170			
HDL cholesterol < 1.02 (mmol/L)	1.909	1.044–3.490	0.036	1.633	0.834–3.197	0.153
Creatinine > 115 (μmol/L)	1.992	0.622–6.378	0.246			
C-reactive protein > 2.0 (mg/L)	1.084	0.600–1.960	0.789			
vWF > median (IU/dl)	5.444	3.062–9.683	< 0.001	2.686	1.424–5.066	0.002
BDNF < median (pg/ml)	7.486	4.077–13.745	< 0.001	2.590	1.287–5.215	0.008

*CAD* coronary artery disease, *LDL* low-density lipoprotein, *HDL* high-density lipoprotein, *vWF* von Willebrand factor, *BDNF* brain-derived neurotrophic factor

**Fig. 1:**
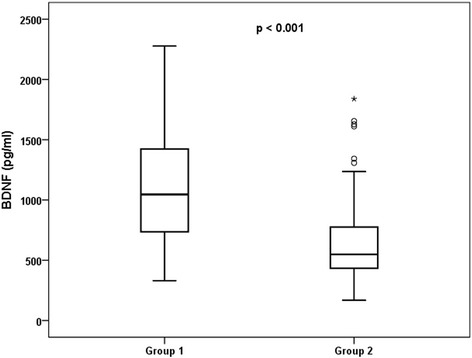
Relationship between plasma BDNF and vWF levels in patients with stable CAD. Group 1 included patients with vWF levels below the median (*n* = 69); Group 2 included patients with vWF levels above the median (*n* = 74). BDFN: brain-derived neurotrophic factor; vWF: von Willebrand factor; CAD, coronary artery disease

We next investigated the correlation between cardiovascular risk factors and plasma BDNF levels in CAD patients (Table 3). The plasma BDNF levels were strongly associated with age (*r* = − 0.274, *p* = 0.001), male sex (*r* = − 0.208, *p* = 0.013), presence of diabetes mellitus (*r* = − 0.421, *p* < 0.001), hypertension (*r* = − 0.220, p = 0.008), LDL cholesterol (*r* = 0.187, *p* = 0.025), HDL cholesterol (*r* = − 0.165, *p* = 0.049), platelet count (*r* = − 0.226, *p* = 0.007) and vWF (*r* = − 0.525, *p* < 0.001, Fig. 1) (Table 3). Multiple linear regression analysis revealed that elderly age, the presence of diabetes mellitus, and vWF levels were independently associated with low BDNF levels in stable CAD patients (Table 4).

**Table 3:** Correlations between cardiovascular risk factors and the plasma level of BDNF in patients with stable CAD (n = 143)

Variable	r	*p* value
Age, years	- 0.274	0.001
Male sex	- 0.208	0.013
Diabetes mellitus	- 0.421	< 0.001
Hypertension	- 0.220	0.008
Body mass index	- 0.053	0.526
Triglycerides	- 0.068	0.422
Total cholesterol	0.019	0.826
LDL cholesterol	0.187	0.025
HDL cholesterol	0.165	0.049
Creatinine	0.097	0.748
Glucose	- 0.030	0.723
C-reactive protein	- 0.047	0.579
Platelet count	0.226	0.007
vWF	- 0.525	< 0.001

*BDNF* brain-derived neurotrophic factor, *CAD* coronary artery disease, *LDL* low-density lipoprotein, *HDL* high-density lipoprotein, *vWF* von Willebrand factor

**Table 4:** Multiple linear regression analysis of cardiovascular risk factor for plasma level of BDNF in patients with stable CAD (n = 143)

	Unstandardized coefficient	Standardized coefficient	*P* value
Age, years	- 0.003	- 0.179	0.008
Male sex	- 0.018	- 0.048	0.466
Diabetes mellitus	- 0.098	- 0.280	< 0.001
Hypertension	- 0.051	- 0.128	0.050
LDL cholesterol	0.096	0.099	0.131
HDL cholesterol	0.105	0.067	0.306
Platelet count	0.159	0.081	0.221
vWF	- 0.003	- 0.407	< 0.001

*BDNF* brain-derived neurotrophic factor, *CAD* coronary artery disease, *LDL* low-density lipoprotein, *HDL* high-density lipoprotein, *vWF* von Willebrand factor. LDL cholesterol, HDL cholesterol and platelet count was logarithm-transformed due to skewed distribution

Subjects were divided into two groups based on the median level of vWF (108 IU/dl) to further evaluate the relationship between BDNF and vWF levels in stable CAD patients. Group 1 included patients with vWF levels below the median, and group 2 included patients with vWF levels above the median. The results showed that BDNF levels were significantly higher in group 1 than group 2 (Fig. 1).

**Fig. 2:**
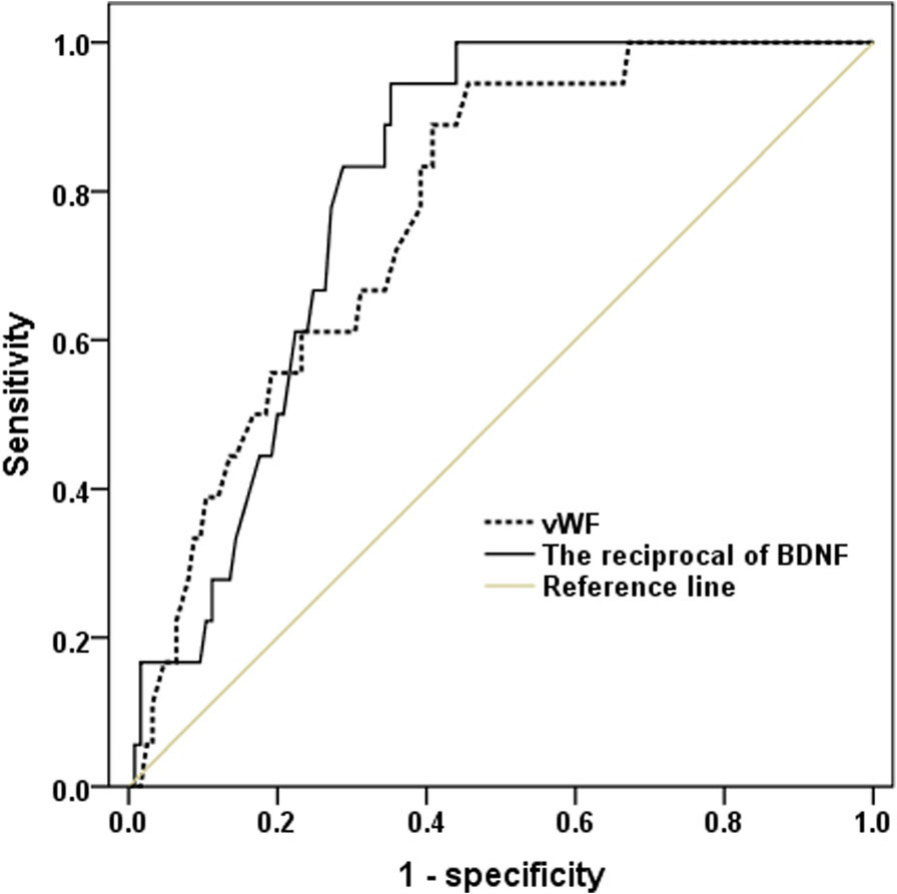
Receiver operator characteristic (ROC) curve for low BDNF levels (AUC 0.804, 95% CI 0.727 to 0.880, *p* < 0.001) and high vWF levels (AUC 0.774, 95% CI 0.678 to 0.870, *p* < 0.001) to detect adverse cardiovascular events in patients with stable CAD. BDNF was transformed reciprocally before ROC analysis. BDFN: brain-derived neurotrophic factor; vWF: von Willebrand factor; CAD, coronary artery disease; AUC: area under the ROC curve

Eighteen patients (12.6%) experienced adverse cardiovascular events after one year of follow-up. The median follow-up time was 11 ± 2 months. Among these patients, one patient had an unexplained sudden death, three patients had an unplanned coronary revascularization, six patients had an ACS, and eight patients had composite of major adverse cardiac events. ROC analysis for the detection of adverse cardiac events revealed an AUC of 0.804 (95% CI: 0.727 to 0.880) for low BDNF versus 0.774 (95% CI: 0.678 to 0.870) for high vWF levels, with no significant difference between the two areas (*p* = 0.747) (Fig. 2). These results indicated that low BDNF and high vWF levels predicted the occurrence of adverse cardiovascular events.

## Discussion

The current study demonstrated the following findings: (1) patients with CAD exhibited lower BDNF levels and higher vWF levels than those of control patients; (2) low BDNF levels were associated with high vWF levels, as an indicator of endothelial dysfunction; (3) the presence of low BDNF and high vWF levels was associated with adverse cardiovascular events at 12-month follow-up in these patients.

Patients with stable CAD exhibited significantly lower BDNF levels compared with those of control patients in this study. Similarly, a small case-control study reported lower plasma levels of BDNF in the ACS patients compared to those of controls [8]. However, the results from different studies may not be directly comparable because of the different populations recruited and observation time points for blood sampling in relation to the index event. Circulating BDNF is produced by the central and peripheral nervous systems [1] and non-neural tissues, including endothelial cells, muscles, and immunocytes [2, 25, 26]. BDNF crosses the blood brain barrier from the brain to the blood [27]. However, serum BDNF levels were not correlated with concurrent brain BDNF levels in rats subjected to hemispheric embolization with microspheres [28]. One study demonstrated a negative correlation between the European cardiovascular risk score and serum BDNF levels in stroke patients [11]. These data indicate that multiple cardiovascular factors strongly influence circulating BDNF levels. We also found that plasma BDNF levels decreased with increasing age and male sex, as reported previously [29]. Low BDNF levels were associated with increased LDL levels and the presence of diabetes mellitus, similar to another study [30]. These results support a potential role for BDNF deficit in the pathogenesis of atherosclerotic cardiovascular disease. Additionally, the present study further suggested that low BDNF levels were associated with high vWF levels even after adjustment for age, LDL levels, gender, and the presence of diabetes mellitus.

vWF levels increase following endothelial damage, and this factor is used as a marker of endothelial dysfunction [19, 31], which is an important early process in atherosclerosis development. Many recent studies have demonstrated that high vWF levels are a powerful prognostic maker in subjects with cardiovascular diseases [20, 21]. Circulating vWF levels are an easy-to-measure, readily available parameter of endothelial dysfunction that requires only blood samples. Therefore, the use of circulating vWF levels to evaluate endothelial function is feasible, effective and practical. The present investigation confirmed that plasma vWF levels were higher in patients with stable CAD than those in controls, which is consistent with previous studies [32–34]. This hypothesis is supported by previous findings indicating that makers for endothelial dysfunction are valuable predictors of the presence and severity of CAD [33]. These results indicate that the endothelial damage involved in the pathogenesis of atherosclerosis may be prevalent in stable CAD patients.

One important finding of our study is that patients with low BDNF levels exhibited increased vWF levels, which potentially reflects endothelial dysfunction. These findings suggest that there is a potential link between endothelial dysfunction and impaired circulating BDNF levels associated with cardiovascular disease. There may be several explanations for this result. First, endothelial cells express BDNF [14]. Second, physical training could increase peripheral BDNF levels in humans [35] and result in enhanced BDNF expression in the endothelium of animals models [14]. Third, hypertension [14] and type 2 diabetes [36] are associated with decreased BDNF expression in the endothelium. Therefore, endothelial function may be an important determinant of circulating BDNF levels in stable CAD patients.

Furthermore, we demonstrated that low BDNF and high vWF levels predicted adverse cardiovascular events at a12-month follow-up in patients with CAD, which is consistent with previous reports [9, 21, 37–40]. Thus, our data suggest the involvement of these two factors in the same mechanism associated with cardiovascular disease. We speculate that endothelial damage reduces the release of BDNF from endothelial cells to the circulation. The biological mechanisms underlying the relationship between low circulating BDNF levels and cardiovascular prognosis are still unclear, but several potential explanations are possible. First, decreased BDNF levels reduce endothelial cell survival and affect angiogenesis [2, 3], which suggested that endothelial dysfunction and low BDNF levels may interact and act as both cause and effect. Second, endothelial BDNF/TrkB signaling protects against atherosclerotic lesion development [41]. Third, BDNF may have a protective anti-inflammatory effect [42]. These results indicated that BDNF has multifaceted cardiovascular protective effects. Therefore, circulating BDNF levels may also be related to vascular endothelial BDNF expression and mirror the cardiovascular status and prognosis. However, further studies are required to validate this hypothesis.

### Study limitations

The results of the present study are encouraging, but there are several limitations that should be acknowledged. First, we had a relatively small study population, and our participants were recruited from a single center within a stable population. Second, circulating BDNF levels are influenced by numerous factors, and not all of the potential determinants were measured in this study. In fact, we excluded some known confounders as possible factors. Third, we did not recruit healthy subjects as controls. Our control subjects were admitted to our hospital with suspected angina pectoris, and they were later confirmed to have no significant coronary stenosis using coronary angiography. Therefore, some control subjects may also have exhibited CAD risk factors. However, these confounding variables do not diminish the value of our results. Finally, we only observed a cross-sectional association between BDNF and vWF levels, and there was a lack of data at multiple time points.

## Conclusions

Plasma BDNF levels in stable CAD patients were inversely associated with vWF levels, and the presence of low BDNF and high vWF levels was predictive of adverse cardiovascular events at a 12-month follow-up. Our data on the association between BDNF and vWF support endothelial dysfunction as an important determinant of low BDNF levels in stable CAD patients. Future studies should determine whether increased BDNF levels are associated with specific treatments for improving endothelial function in these patients.

## Abbreviations

ACEI: Angiotensin-converting enzyme inhibitor
ACS: Acute coronary syndrome
ARB: Angiotensin receptor blocker
BDNF: Brain-derived neurotrophic factor;
CABG: Coronary artery bypass grafting
CAD: Coronary artery disease
CCB: Calcium channel blocker
CI: Confidence intervals
EF: Ejection fraction
ELISA: Enzyme-linked immunosorbent assay
HDL: High-density lipoprotein
LDL: Low-density lipoprotein
MI: Myocardial infarction
OR: Odds ratios
PCI: Percutaneous coronary intervention
ROC: Receiver operating characteristic
STEMI: ST segment elevation myocardial infarction
TrkB: Tyrosine receptor kinase B
vWF: von Willebrand factor
